# Quercetin Suppresses Uterine Leiomyoma Progression by Modulating METTL3-Mediated MAPK Signaling

**DOI:** 10.3390/ijms27104586

**Published:** 2026-05-20

**Authors:** Wenting Luo, Xuan Yang, Yu Liu, Tiantian Qiu, Hui Ren, Jiheng Zuo, Zongshun Chen, Shuoshuo Shi, Donghua Li

**Affiliations:** 1 School of Traditional Chinese Medicine, Capital Medical University, Beijing 100069, China; 2 Experimental Research Center, China Academy of Chinese Medical Sciences, Beijing 100700, China; 3 Institute of Basic Research in Clinical Medicine, China Academy of Chinese Medical Sciences, Beijing 100700, China

**Keywords:** quercetin, uterine leiomyoma, METTL3, MAPK signaling, proliferation, apoptosis, inflammation

## Abstract

Uterine leiomyoma (UL) is characterized by excessive proliferation, extracellular matrix accumulation, and inflammatory activation, yet its upstream regulatory mechanisms remain incompletely defined. Here, we investigated the role of METTL3-associated signaling in mediating the anti-leiomyoma effects of quercetin. Quercetin significantly inhibited proliferation and induced apoptosis in UL cells, accompanied by suppression of inflammatory cytokine production. Transcriptomic profiling revealed that METTL3 silencing was associated with enrichment of MAPK and inflammation-related pathways. Mechanistically, quercetin downregulated METTL3 expression and suppressed phosphorylation of MEK, ERK, JNK, and p38, whereas METTL3 overexpression partially reversed these effects, supporting a functional role of METTL3 in mediating MAPK pathway activation. Consistently, METTL3 knockdown recapitulated the anti-proliferative, pro-apoptotic, and anti-inflammatory effects of quercetin. In a hormone-induced UL rat model, quercetin attenuated uterine enlargement, fibrosis, and proliferative activity, accompanied by decreased METTL3 expression and MAPK activation. Collectively, these findings demonstrate that quercetin suppresses UL progression, at least in part, through modulation of METTL3-mediated MAPK signaling, highlighting METTL3 as a critical regulatory node and a potential therapeutic target in UL.

## 1. Introduction

Uterine leiomyoma (UL) is the most prevalent benign tumor of the female reproductive system and represents a major gynecological disorder in women of reproductive age [1,2]. Epidemiological studies based on ultrasound screening estimate that the cumulative incidence of UL reaches approximately 70–80% by the age of 50 in several populations [3]. Although many lesions remain clinically silent, nearly one-third of patients develop symptoms such as abnormal uterine bleeding, pelvic pain, infertility, and recurrent pregnancy loss [4,5,6]. At the pathological level, UL is characterized by dysregulated proliferation of uterine smooth muscle cells, excessive extracellular matrix deposition, and progressive fibrotic remodeling [7,8]. These processes are driven by a complex microenvironment involving inflammatory mediators, growth factors, and mechanotransduction signals that collectively promote tumor growth and persistence [9,10]. Current therapeutic strategies, including surgical intervention and hormone-based treatments, are often limited by adverse endocrine effects and high recurrence rates [11,12,13]. Therefore, identifying mechanism-based therapeutic targets that address the molecular drivers of leiomyoma progression remains a critical unmet need in UL management.

Natural compounds with multi-target regulatory properties have emerged as promising modulators of leiomyoma-related signaling networks [14]. Quercetin, a widely distributed flavonoid, exhibits anti-proliferative, anti-inflammatory, antioxidant, and anti-fibrotic activities across diverse pathological conditions [15,16,17]. In cancer models, quercetin suppresses cell proliferation and induces apoptosis through modulation of signaling pathways including MAPK, NF-κB, and PI3K/AKT [17]. It has also been reported to attenuate fibrotic remodeling and inflammatory signaling in hepatic and pulmonary fibrosis by suppressing MAPK activation and regulating apoptosis-related proteins [18]. Notably, MAPK signaling plays a central role in UL pathogenesis by regulating cell proliferation, extracellular matrix production, and inflammatory responses [19,20].

Activation of ERK, JNK, and p38 pathways contributes to leiomyoma development, while upstream factors such as transforming growth factor-β and gonadotropin-releasing hormone further enhance MAPK-dependent extracellular matrix accumulation [21,22]. Similarly, activin A promotes leiomyoma proliferation and extracellular matrix production via p38 MAPK signaling [23]. In parallel, inflammatory cytokines including IL-6 and IL-8 are elevated in leiomyoma tissues and promote proliferative and fibrotic responses [24]. Despite these insights, the upstream molecular mechanisms by which quercetin modulates these signaling networks in UL remain poorly defined.

METTL3 has recently emerged as a critical regulator of cellular signaling programs associated with proliferation, inflammation, and tumor progression. Although METTL3 was initially characterized as the catalytic component of the N6-methyladenosine (m6A) methyltransferase complex responsible for RNA modification [25], accumulating evidence indicates that its functions extend beyond canonical methyltransferase activity. For instance, METTL3 can promote the translation of oncogenic transcripts through interactions with the translational machinery independently of m6A modification [26], and cooperate with eIF3h to enhance mRNA circularization and translation efficiency [27]. In addition, cytoplasmic METTL3 has been shown to sustain aberrant signaling programs in both hematologic malignancies and solid tumors, even in the absence of its enzymatic activity [26,28]. More importantly, recent studies have demonstrated that METTL3 participates in the regulation of inflammatory gene expression and stress responses independently of m6A modification, linking it to broader transcriptional and signaling networks [29,30,31]. Notably, transcriptomic analyses have shown that METTL3 perturbation frequently affects pathways associated with proliferation, inflammation, and MAPK signaling [32,33]. These findings suggest that METTL3 may function as a key regulatory hub coordinating multiple disease-relevant signaling pathways, rather than acting solely as an RNA methylation enzyme.

Based on these observations, we hypothesized that quercetin suppresses UL progression through modulation of METTL3-associated signaling pathways. In this study, we demonstrate that quercetin inhibits leiomyoma cell proliferation and promotes apoptosis by suppressing METTL3-dependent MAPK signaling. Using integrated in vitro and in vivo approaches combined with transcriptomic profiling and genetic manipulation of METTL3, we further establish METTL3 as a key mediator of quercetin-induced anti-proliferative, pro-apoptotic, and anti-inflammatory effects.

## 2. Results

### 2.1. Quercetin Suppresses Proliferation and Induces Apoptosis in UL Cells

**Figure 1:**
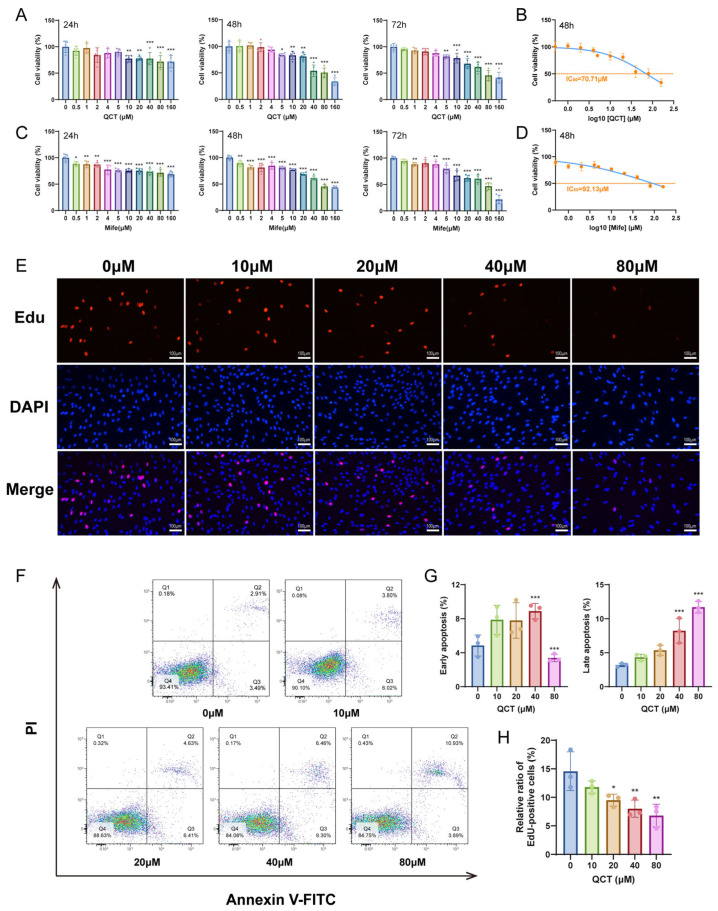
Quercetin suppresses proliferation and promotes apoptosis in UL cells. (**A**,**C**) UL cells were exposed to increasing concentrations of QCT (0–160 μM) or Mife (0–160 μM) for 24, 48, and 72 h. Cell viability was determined using the CCK-8 assay. (**B**,**D**) Dose–response curves and calculated IC_50_ values of QCT and Mife at 48 h. (**E**,**H**) EdU incorporation assay showing cell proliferation after QCT treatment (0, 10, 20, 40, and 80 μM for 48 h). Representative fluorescence images (200×), with red indicating EdU-positive cells and blue indicating DAPI-stained nuclei, and quantification of EdU-positive cells (*n* = 3). (**F**,**G**) Apoptosis analysis by Annexin V-FITC/PI flow cytometry following QCT treatment for 48 h, with representative plots and quantification of early and late apoptotic cells (*n* = 3). The gating strategy and control samples are shown in Supplementary Figure S1. Data are presented as mean ± SD. * *p* < 0.05, ** *p* < 0.01, *** *p* < 0.001 vs. control (0 μM).

Cell viability was first evaluated using CCK-8 assays. Quercetin (QCT) treatment markedly reduced the viability of UL cells, with stronger inhibitory effects observed at higher concentrations and extended treatment periods (Figure 1A). A comparable inhibitory trend was observed with the positive control drug mifepristone (Mife) (Figure 1C). Notably, the inhibitory effect of quercetin became more prominent at 48 h and plateaued thereafter, suggesting that 48 h represents a stable pharmacological response window. Dose–response analysis further indicated that the half-maximal inhibitory concentrations (IC_50_) at 48 h were 70.71 μM for quercetin and 92.13 μM for mifepristone, respectively (Figure 1B,D).

To determine whether the reduced cell viability was associated with impaired proliferative capacity, EdU incorporation assays were performed. Quercetin treatment led to a progressive decline in EdU-positive cells, indicating a dose-dependent suppression of DNA synthesis and proliferative activity in UL cells (Figure 1E,H).

In agreement with these findings, Annexin V-FITC/PI flow cytometric analysis showed that quercetin significantly increased both early and late apoptotic cell populations compared with controls (Figure 1F,G). Specifically, early apoptotic cells increased at lower to moderate concentrations of quercetin, whereas higher concentrations predominantly promoted late apoptosis. Notably, the proportion of late apoptotic cells exhibited a clear dose-dependent increase, indicating progressive apoptotic cell death. These results suggest that quercetin reduces UL cell viability at least in part by inducing apoptosis, rather than solely inhibiting proliferation.

Considering the dose–response characteristics, IC_50_ estimation, and cellular tolerance, 20 μM and 40 μM quercetin were chosen to represent the low and high concentration treatments, respectively, with 48 h as the standard treatment duration for subsequent mechanistic experiments. In parallel, 20 μM mifepristone for 48 h was used as the positive control condition.

### 2.2. Establishment and Validation of METTL3 Knockdown and Overexpression Models in UL Cells

**Figure 2:**
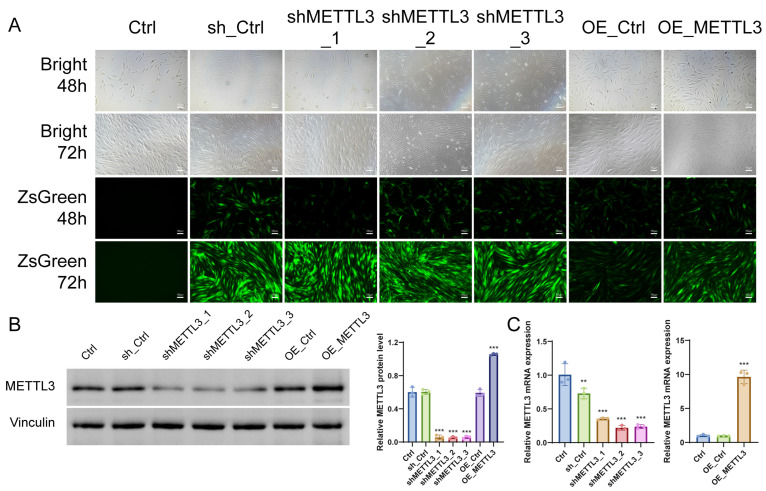
Establishment of METTL3 knockdown and overexpression models in UL cells. (**A**) Representative bright-field and ZsGreen fluorescence images of UL cells after lentiviral transduction (48 h and 72 h, 100× magnification). (**B**) Representative Western blot images and quantitative analysis of METTL3 protein expression. (**C**) RT-qPCR analysis of METTL3 mRNA expression in METTL3 knockdown and overexpression cells. Data are presented as mean ± SD (*n* = 3). ** *p* < 0.01, *** *p* < 0.001 vs. control.

To explore the potential molecular mechanisms underlying the anti-proliferative and pro-apoptotic effects of quercetin in UL cells, METTL3 expression was genetically manipulated using lentiviral transduction. Bright-field and fluorescence imaging confirmed efficient transduction without obvious cytotoxicity (Figure 2A).

Western blotting and RT-qPCR further verified successful modulation of METTL3 expression at both the protein and mRNA levels (Figure 2B,C). Among the three knockdown constructs, shMETTL3_1 exhibited the most stable and efficient silencing effect and was therefore selected for subsequent experiments.

### 2.3. METTL3 Regulates Disease-Related Transcriptional Programs in UL Cells

**Figure 3:**
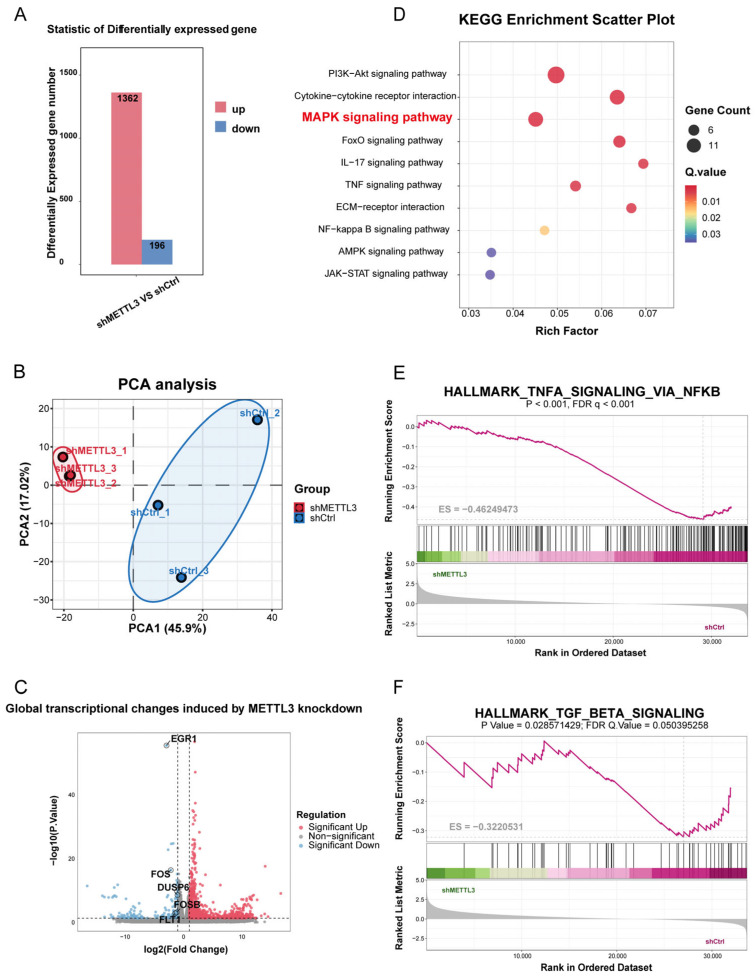
Transcriptomic profiling of METTL3 knockdown in UL cells. (**A**) Overview of differentially expressed genes identified by RNA-seq analysis. (**B**) PCA showing sample clustering between groups. (**C**) Volcano plot showing global transcriptional changes induced by METTL3 knockdown. (**D**) KEGG pathway enrichment analysis of differentially expressed genes highlighting key signaling pathways. (**E**,**F**) Gene set enrichment analysis (GSEA) demonstrating enrichment of TNFα signaling via NF-κB and TGF-β signaling pathways.

To delineate the downstream transcriptional programs regulated by METTL3 in UL cells, RNA sequencing was performed in shMETTL3 and shCtrl cells. PCA analysis showed clear separation between the two groups, indicating high reproducibility and a distinct global transcriptional shift after METTL3 silencing (Figure 3B). Differential expression analysis identified 1362 upregulated and 196 downregulated genes in shMETTL3 cells compared with shCtrl cells, indicating a substantial regulatory role of METTL3 in ULM cells (Figure 3A,C).

Functional enrichment analysis revealed that METTL3 knockdown predominantly affected pathways associated with proliferative signaling, inflammatory responses, and microenvironmental remodeling, processes closely related to UL progression. KEGG analysis showed enrichment of several representative pathways, including MAPK signaling, PI3K-Akt signaling, cytokine–cytokine receptor interaction, and TNF signaling (Figure 3D). Consistently, GSEA further identified significant enrichment of TNFα signaling via NF-κB and TGF-β signaling in the METTL3-associated transcriptional profile (Figure 3E,F), supporting a role for METTL3 in inflammatory activation and extracellular matrix remodeling in UL cells.

Collectively, these findings suggest that METTL3 orchestrates a broad disease-related transcriptional network rather than a single downstream event. Given its prominent enrichment and its direct relevance to the anti-proliferative and pro-apoptotic phenotype induced by quercetin, the MAPK pathway was selected for subsequent mechanistic validation.

### 2.4. METTL3 Mediates the Inhibitory Effect of Quercetin on MAPK Signaling and Inflammatory Responses in UL Cells

To determine whether METTL3 mediates the effects of quercetin on MAPK signaling, METTL3 knockdown and overexpression models were combined with quercetin treatment in UL cells.

**Figure 4:**
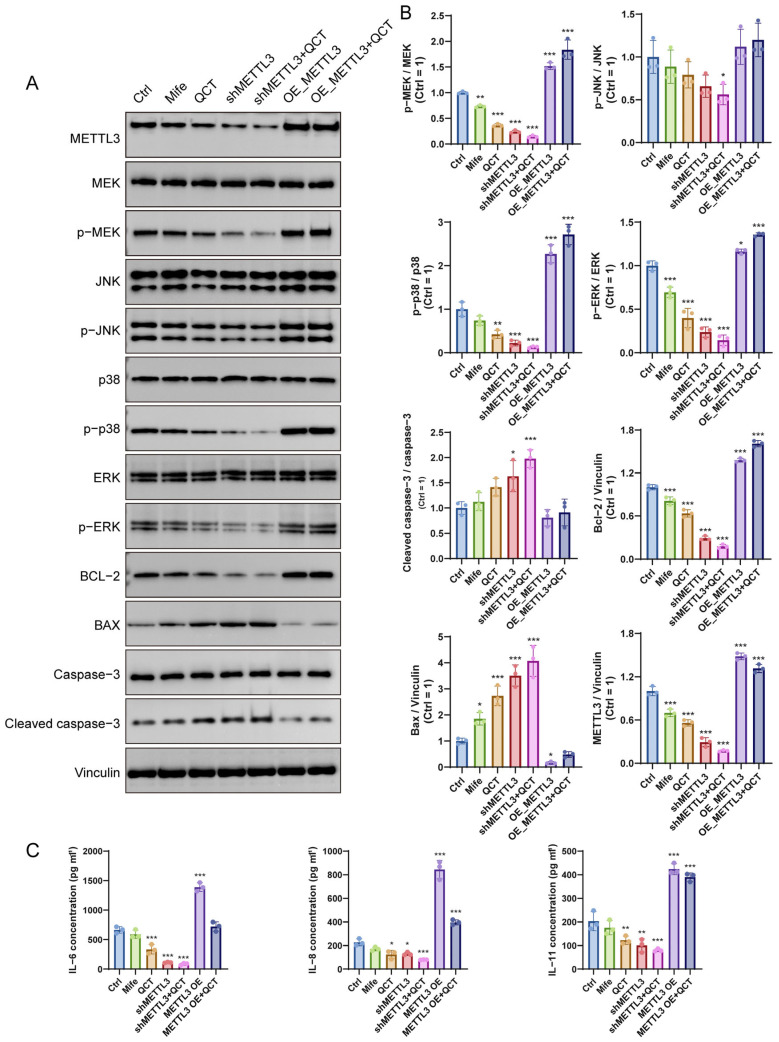
QCT regulates MAPK signaling and apoptosis through METTL3 in UL cells. (**A**,**B**) Western blot analysis of METTL3 expression, MAPK pathway activation (p-MEK/MEK, p-ERK/ERK, p-JNK/JNK, p-p38/p38), and apoptosis-related proteins (Bcl-2, Bax, cleaved caspase-3/caspase-3), with corresponding quantitative analysis. (**C**) ELISA analysis of inflammatory cytokines (IL-6, IL-8, and IL-11). * *p* < 0.05, ** *p* < 0.01, *** *p* < 0.001 vs. control.

Western blot analysis showed that quercetin downregulated METTL3 expression and markedly decreased the phosphorylation levels of MEK, ERK, JNK, and p38 without affecting total protein levels, indicating suppression of MAPK pathway activation (Figure 4A,B). A similar inhibitory pattern was observed in shMETTL3 cells, whereas METTL3 overexpression enhanced MAPK phosphorylation and largely attenuated the suppressive effect of quercetin, supporting a functional association between METTL3 and MAPK pathway activation in the context of quercetin treatment.

Consistent with MAPK inhibition, both quercetin treatment and METTL3 silencing promoted a pro-apoptotic protein profile, characterized by increased Bax and cleaved caspase-3, together with decreased Bcl-2 expression (Figure 4A,B). In contrast, METTL3 overexpression attenuated the pro-apoptotic effect induced by quercetin. In parallel, ELISA analysis showed that quercetin significantly reduced the secretion of IL-6, IL-8, and IL-11. Similar reductions were observed following METTL3 silencing, whereas METTL3 overexpression partially restored cytokine production (Figure 4C). Taken together, these findings indicate that quercetin suppresses MAPK activation, at least in part in association with METTL3 modulation.

### 2.5. Quercetin Attenuates UL Progression In Vivo

**Figure 5:**
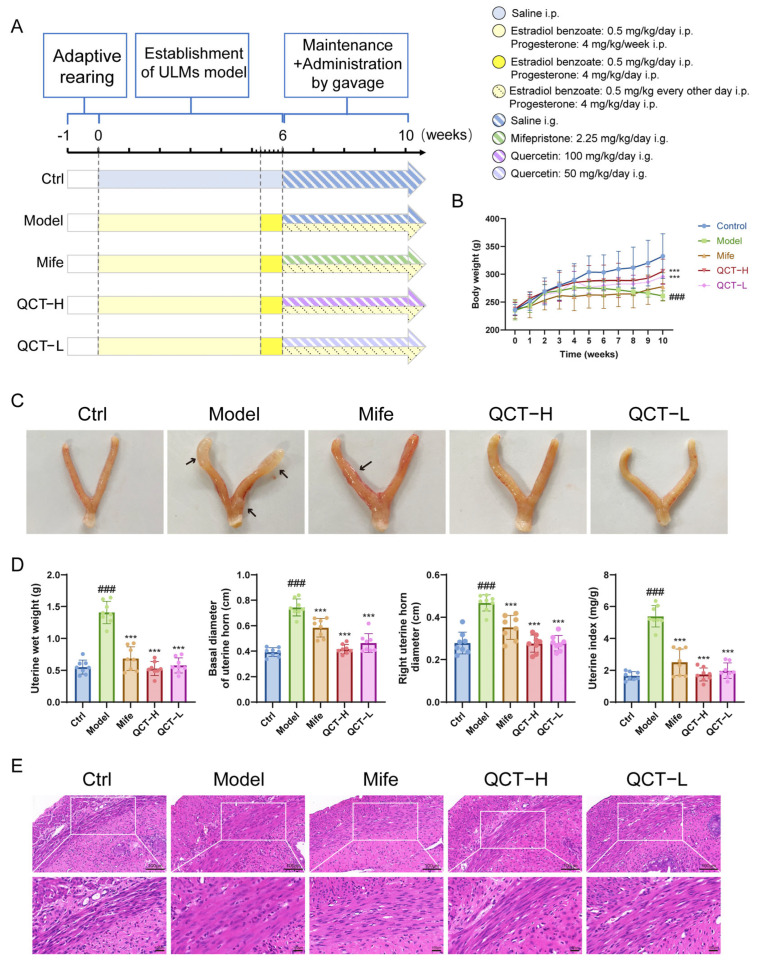
QCT attenuates UL progression in vivo. (**A**) Schematic diagram of the experimental design and treatment schedule. (**B**) Body weight changes in rats during the experimental period (*n* = 8). (**C**) Representative images of rat uteri from each group. (**D**) Quantitative analysis of uterine wet weight, basal diameter of uterine horn, right uterine horn diameter, and uterine index (*n* = 8). (**E**) Representative H&E-stained sections of uterine tissues (400×). Data are presented as mean ± SD. ### *p* < 0.001 vs. control. *** *p* < 0.001 vs. model.

To evaluate the therapeutic efficacy of quercetin in vivo, a hormone-induced UL rat model was established followed by oral administration of quercetin or mifepristone according to the experimental schedule (Figure 5A). Body weight increased steadily in all groups during the experimental period, and no obvious systemic toxicity was observed in quercetin-treated rats (Figure 5B).

Gross anatomical examination showed that the model group developed enlarged and irregular uterine horns, whereas treatment with quercetin or mifepristone markedly alleviated these morphological alterations (Figure 5C). Consistently, quantitative measurements demonstrated that uterine wet weight, basal uterine horn diameter, right uterine horn diameter, and uterine index were all significantly elevated in the model group compared with the control group. Both high and low doses of quercetin effectively reduced these parameters, showing comparable or stronger inhibitory effects than mifepristone (Figure 5D).

Histological evaluation further supported these observations. Hematoxylin and eosin staining showed normal uterine architecture in the control group, with orderly arranged smooth muscle bundles and minimal connective tissue. In contrast, the model group exhibited typical pathological features of uterine leiomyoma, including smooth muscle cell hypertrophy, disorganized and densely packed muscle bundles, myometrial thickening, and increased connective tissue deposition. Treatment with quercetin or mifepristone markedly attenuated these abnormalities, resulting in a more regular smooth muscle arrangement, reduced cellular hypertrophy, decreased myometrial thickness, and diminished connective tissue accumulation. These findings indicate that quercetin effectively improves the histopathological alterations associated with uterine leiomyoma. (Figure 5E).

Taken together, these results demonstrate that quercetin effectively suppresses UL progression in vivo and improves pathological alterations in uterine tissue. Both 50 mg/kg and 100 mg/kg quercetin produced significant therapeutic effects, with the higher dose generally showing more pronounced improvements, indicating an overall dose-dependent trend.

### 2.6. Quercetin Suppresses Collagen Deposition, Proliferation, and MAPK Signaling In Vivo

**Figure 6:**
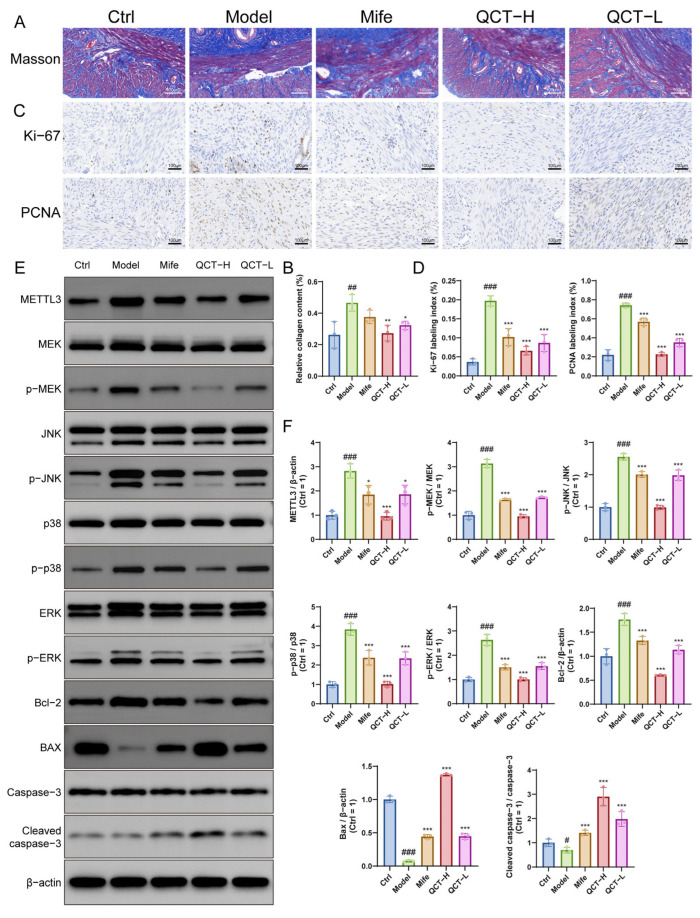
QCT inhibits fibrosis, proliferation, and MAPK signaling in vivo. (**A**,**B**) Masson’s trichrome staining showing collagen deposition and quantification of relative collagen content (400×, *n* = 3). (**C**,**D**) Immunohistochemical staining of Ki-67 and PCNA with quantification (600×, *n* = 3). (**E**,**F**) Western blot analysis of METTL3 expression, MAPK pathway activation (p-MEK/MEK, p-ERK/ERK, p-JNK/JNK, p-p38/p38), and apoptosis-related proteins (Bcl-2, Bax, cleaved caspase-3/caspase-3), with corresponding quantitative analysis (*n* = 3). Data are presented as mean ± SD. # *p* < 0.05, ## *p* < 0.01, ### *p* < 0.001 vs. control. * *p* < 0.05, ** *p* < 0.01, *** *p* < 0.001 vs. model.

Masson’s trichrome staining revealed pronounced collagen deposition in the model group, indicating excessive extracellular matrix accumulation, whereas quercetin treatment significantly reduced collagen deposition in uterine tissues (Figure 6A,B). Consistently, immunohistochemistry showed that the proliferation markers Ki-67 and PCNA were markedly elevated in the model group but were significantly decreased following quercetin administration (Figure 6C,D), indicating inhibition of UL-associated proliferative activity in vivo.

At the molecular level, Western blot analysis showed that the model group exhibited increased METTL3 expression and enhanced phosphorylation of MAPK pathway components including MEK, ERK, JNK, and p38. Quercetin treatment markedly reduced METTL3 expression and suppressed MAPK phosphorylation without affecting total protein levels (Figure 6E,F). In parallel, quercetin shifted apoptosis-related protein expression toward a pro-apoptotic profile, characterized by decreased Bcl-2 and increased Bax and cleaved caspase-3 levels.

Collectively, these findings demonstrate that quercetin attenuates collagen deposition and fibrosis-like histopathological changes, suppresses abnormal proliferation while promoting apoptosis in vivo, which is accompanied by modulation of the METTL3- associated MAPK signaling pathway. These effects were observed at both tested doses and were generally more pronounced in the 100 mg/kg group, further supporting a dose-dependent therapeutic effect of quercetin in vivo.

## 3. Discussion

Uterine leiomyoma (UL) is characterized by dysregulated proliferation, excessive extracellular matrix deposition, and a chronic inflammatory microenvironment that collectively drive fibroid growth and tissue remodeling. Although multiple signaling pathways have been implicated in these processes, the upstream regulatory mechanisms coordinating these events remain incompletely defined. In this study, we demonstrate that quercetin suppresses UL progression through modulation of a METTL3-associated MAPK signaling axis. By integrating functional assays, transcriptomic profiling, and genetic manipulation of METTL3, our findings identify METTL3 as a critical regulatory node linking proliferative signaling, apoptotic balance, and inflammatory responses in leiomyoma cells. Importantly, these effects were consistently observed in both in vitro and in vivo models, supporting the robustness of this regulatory mechanism.

Quercetin has been widely reported to exert anti-proliferative, anti-inflammatory, and anti-fibrotic effects across diverse disease models. Previous studies have shown that quercetin modulates kinase-driven signaling pathways, including MAPK and PI3K/AKT cascades, thereby inhibiting tumor growth and inflammatory activation [15,18,34]. In fibrotic diseases, quercetin has been reported to reduce extracellular matrix deposition and inflammatory cytokine production through inhibition of stress-activated signaling pathways [17,35]. Notably, the doses used in this study (50 and 100 mg/kg) have been shown to be effective in multiple rat models, where quercetin attenuated inflammatory responses, oxidative stress, and collagen deposition, indicating that our dosing regimen falls within a biologically relevant and well-established therapeutic range [36,37]. Consistent with these reports, both doses significantly improved uterine morphology, reduced collagen deposition, suppressed abnormal proliferation, and modulated apoptosis- and METTL3/MAPK-related signaling in vivo, with the 100 mg/kg dose generally producing more pronounced effects. Consistent with these findings, our results demonstrate that quercetin significantly inhibits MAPK activation in UL cells, leading to reduced proliferation and increased apoptosis. Given the central role of MAPK signaling in regulating fibroid growth and extracellular matrix remodeling, these findings further support the therapeutic potential of quercetin in UL.

A key mechanistic insight of this study is the identification of METTL3 as an upstream regulator linking quercetin treatment to MAPK pathway modulation. While METTL3 has been extensively studied as a core component of the m6A RNA methylation machinery, emerging evidence suggests that it also participates in translational regulation and signaling control independent of its canonical enzymatic function [25,38]. In this context, our results suggest that METTL3 may influence MAPK signaling by coordinating the expression or translation of key upstream regulators involved in kinase activation and inflammatory signaling. Supporting this notion, METTL3 knockdown recapitulated the inhibitory effects of quercetin, whereas METTL3 overexpression partially reversed these effects, indicating that METTL3 is functionally required for MAPK pathway activation in UL cells. These findings position METTL3 as a central signaling hub that integrates epitranscriptomic regulation with MAPK-dependent cellular responses.

Importantly, these effects were consistently observed in both in vitro and in vivo models, supporting the robustness of this regulatory mechanism. However, it should be noted that quercetin was directly applied to cultured UL cells in vitro, whereas it was administered orally in vivo. Therefore, the observed in vivo effects may be mediated not only by the parent compound but also by its intestinal and hepatic metabolites. In a hormone-induced UL rat model, quercetin administration significantly attenuated uterine enlargement, collagen deposition, and proliferative activity. These phenotypic improvements were accompanied by decreased METTL3 expression and suppression of MAPK pathway activation, consistent with our in vitro observations. Taken together, our findings suggest that quercetin inhibits UL progression by downregulating METTL3, thereby suppressing MAPK signaling and its downstream effects on proliferation, apoptosis, and inflammatory responses.

Several limitations should be acknowledged. First, although our study establishes a functional association between METTL3 and MAPK signaling, the precise molecular interactions underlying this regulation remain to be elucidated. Future studies are needed to determine whether METTL3 directly regulates specific upstream kinases or signaling intermediates. Second, UL is a multifactorial disease involving complex hormonal and microenvironmental interactions, and additional pathways may contribute to the observed effects. Third, due to metabolic transformation following oral administration, the active molecular species responsible for the in vivo effects of quercetin remain to be fully characterized. Finally, further investigation of the pharmacokinetics, bioavailability, and optimal dosing strategies of quercetin is warranted to facilitate its potential clinical application.

## 4. Materials and Methods

### 4.1. Reagents

Quercetin (≥95% purity; Cat. No. Q4951) was purchased from Sigma-Aldrich (St. Louis, MO, USA). Mifepristone (Cat. No. S25748) was obtained from Shanghai Yuanye Bio-Technology Co., Ltd. (Shanghai, China). Dulbecco’s Modified Eagle Medium (DMEM; Cat. No. 12320032), fetal bovine serum (FBS; Cat. No. A5669701), and penicillin–streptomycin (100×; Cat. No. 15140122) were purchased from Gibco (Thermo Fisher Scientific, Waltham, MA, USA). Estradiol benzoate injection and progesterone injection were purchased from Sichuan Jinke Pharmaceutical Co., Ltd. (Chengdu, China). 4% paraformaldehyde fix solution (Cat. No. P0099), Triton X-100 (Cat. No. RH72715), and anti-fade mounting medium with DAPI (Cat. No. P0131) were purchased from Beyotime Biotechnology (Shanghai, China). Trypsin solution without EDTA (Cat. No. GP3190) was obtained from Genview (Beijing, China). Masson’s trichrome staining kit (Cat. No. G1006) was purchased from Beijing Solarbio Science & Technology Co., Ltd. (Beijing, China).

### 4.2. Cell Culture

Human uterine leiomyoma cells (Cat. No. CP-H151, Procell Life Science & Technology, Wuhan, China) were cultured in DMEM supplemented with 10% FBS and 1% penicillin–streptomycin. Cells were maintained at 37 °C in a humidified incubator containing 5% CO_2_ and passaged at 70–80% confluence.

### 4.3. Cell Viability Assay (CCK-8)

Cell viability was assessed using the Cell Counting Kit-8 (CCK-8; Cat. No. C0043, Beyotime Biotechnology, Shanghai, China) according to the manufacturer’s protocol. UL cells were seeded into 96-well plates at a density of 5 × 10^3^ cells per well and allowed to adhere overnight. Cells were then treated with increasing concentrations of quercetin or mifepristone (0–160 μM) for 24, 48, or 72 h. Subsequently, 10 μL of CCK-8 reagent was added to each well containing 100 μL of culture medium and incubated for 2 h at 37 °C. Absorbance was measured at 450 nm using a microplate reader (SpectraMax iD3, Molecular Devices, San Jose, CA, USA).

### 4.4. EdU Cell Proliferation Assay

Cell proliferation was determined using the BeyoClick™ EdU-594 Cell Proliferation Detection Kit (Cat. No. C0078L, Beyotime Biotechnology, Shanghai, China). Following treatment, UL cells were incubated with EdU, fixed, permeabilized, and stained with Click reaction solution, followed by DAPI counterstaining. Fluorescence images were captured using a fluorescence microscope (Nikon ECLIPSE Ti-U, Nikon Corporation, Tokyo, Japan). The proliferation rate was calculated as the percentage of EdU-positive cells relative to the total DAPI-stained nuclei using ImageJ software (version 1.54g, NIH, Bethesda, MD, USA).

### 4.5. Flow Cytometric Analysis of Apoptosis

Cell apoptosis was analyzed using an Annexin V-FITC/PI Apoptosis Detection Kit (Cat. No. C1062M, Beyotime Biotechnology, Shanghai, China) according to the manufacturer’s protocol. After treatment, UL cells were harvested by trypsinization without EDTA and washed twice with cold PBS. Cells were resuspended in binding buffer and incubated with Annexin V-FITC and propidium iodide (PI) for 20–30 min in the dark at room temperature. Apoptotic cells were analyzed using a flow cytometer (LSRFortessa, Becton Dickinson, San Jose, CA USA). For flow cytometric analysis, cells were first gated on FSC-A versus SSC-A to exclude debris, followed by FSC-A versus FSC-H to select single cells. Compensation was performed using unstained and single-stained controls (Annexin V-FITC or PI). Apoptotic populations were identified by quadrant gating based on Annexin V-FITC and PI fluorescence signals.

### 4.6. RNA Extraction and Quantitative Real-Time PCR

Total RNA was extracted using the FastPure Cell/Tissue Total RNA Isolation Kit V2 (Cat. No. RC112, Vazyme Biotech Co., Ltd., Nanjing, China). RNA concentration and purity were assessed using a micro-volume spectrophotometer (NanoDrop, Thermo Fisher Scientific, Waltham, MA, USA). cDNA was synthesized using HiScript III RT SuperMix for qPCR (+gDNA Wiper) (Cat. No. Q711, Vazyme Biotech Co., Ltd., Nanjing, China). Quantitative real-time PCR was conducted using ChamQ Universal SYBR qPCR Master Mix (Cat. No. Q311, Vazyme Biotech Co., Ltd., Nanjing, China) on a CFX96 Real-Time PCR Detection System (Bio-Rad Laboratories, Hercules, CA, USA). Relative gene expression levels were calculated using the 2^−^ΔΔCt method with GAPDH as the internal reference gene. Primer sequences are listed in Supplementary Table S1.

### 4.7. Western Blot Analysis

Total proteins from UL cells or rat uterine tissues were extracted and quantified using the BCA Protein Assay Kit (Cat. No. WB6501, NCM Biotech, Suzhou, China). Equal amounts of protein were separated by SDS-PAGE along with a prestained protein molecular weight marker (Prestained Protein Marker VII, 8–195 kDa, Cat. No. G2087-250UL, Servicebio, Wuhan, China) and transferred onto PVDF membranes (Cat. No. IPVH00010, Millipore, Billerica, MA, USA). Membranes were blocked with 5% non-fat milk and incubated overnight at 4 °C with primary antibodies against MEK (11049-1-AP), p-MEK (81304-1-RR), JNK (66210-1-Ig), p-JNK (80024-1-RR), p38 (14064-1-AP), p-p38 (28796-1-AP), ERK (11257-1-AP), p-ERK (28733-1-AP), and Vinculin (26520-1-AP) (all from Proteintech, Wuhan, China); METTL3 (ab195352), BCL-2 (ab196495), BAX (ab32503) and β-Actin (ab8226) (all from Abcam, Cambridge, UK); cleaved caspase-3 (9661S) and caspase-3 (9662S) (both from Cell Signaling Technology, Danvers, MA, USA). After washing with TBST, membranes were incubated with HRP-conjugated secondary antibodies for 1 h at room temperature. Protein bands were visualized using an enhanced chemiluminescence kit (Cat. No. GE2301, Genview, Beijing, China) and imaged using a Bio-Rad ChemiDoc system. Band intensities were quantified using ImageJ software (version 1.54g, NIH, Bethesda, MD, USA), with Vinculin or β-Actin as loading controls.

### 4.8. Lentiviral-Mediated METTL3 Overexpression and Knockdown

METTL3 expression was modulated by lentiviral transduction. For knockdown, three short hairpin RNAs targeting METTL3 (shMETTL3-1, shMETTL3-2, and shMETTL3-3) and a non-targeting control (shCtrl) were constructed (sequences provided in Supplementary Table S2). For overexpression, a lentiviral vector encoding full-length METTL3 (NM_019852.5) and its corresponding empty vector control (OE-Control) were used. Lentiviral constructs were purchased from GeneChem (Shanghai, China). UL cells were infected according to the manufacturer’s protocol and selected with puromycin to establish stable METTL3 knockdown or overexpression cell lines. The efficiency of METTL3 knockdown and overexpression was verified by quantitative real-time PCR and Western blotting.

### 4.9. RNA Sequencing (RNA-Seq) and Bioinformatics Analysis

Total RNA from shMETTL3 and shCtrl cells (*n* = 3 per group) was extracted and evaluated using a NanoDrop spectrophotometer (Thermo Fisher Scientific, Wilmington, DE, USA) and Agilent 2100 Bioanalyzer (Agilent Technologies, Santa Clara, CA, USA). RNA library construction and sequencing were performed by LC-Bio Technology Co., Ltd. (Hangzhou, China). Poly(A)+ mRNA was enriched and used for cDNA library preparation. Sequencing was conducted on the Illumina NovaSeq 6000 platform. Clean reads were aligned to the human reference genome using HISAT2, and gene expression levels were quantified as FPKM values. Differentially expressed genes (DEGs) were identified using DESeq2 with thresholds of |log_2_ fold change| ≥ 1 and adjusted *p* < 0.05.

Functional enrichment analyses, including Kyoto Encyclopedia of Genes and Genomes (KEGG) pathway analysis and Gene Set Enrichment Analysis (GSEA), were performed to identify significantly enriched pathways. All bioinformatics analyses were performed using the LC-Bio Cloud Platform.

### 4.10. Enzyme-Linked Immunosorbent Assay (ELISA)

The concentrations of secreted IL-6, IL-8, and IL-11 in cell culture supernatants were measured using ELISA kits (IL-6: CSB-E04638h-IS; IL-8: CSB-E04641h; IL-11: CSB-E04596h; CUSABIO Biotech, Wuhan, China) according to the manufacturer’s instructions. Absorbance was measured at 450 nm using a microplate reader, and cytokine concentrations were calculated from standard curves.

### 4.11. Animal Model Establishment and Treatment

Female Sprague–Dawley rats (2–3 months old, 220 ± 10 g) were obtained from Vital River Laboratory Animal Technology Co., Ltd. (Beijing, China) and housed under specific pathogen-free conditions (24 ± 1 °C, 50% humidity, 12 h light/dark cycle) with free access to food and water. All procedures were approved by the Institutional Animal Care and Use Committee of Capital Medical University (Ethics No. AEEI-2022-295) and performed in accordance with ARRIVE guidelines.

After a 1-week acclimatization period, rats were randomly divided into five groups (*n* = 8 per group): control (Ctrl), model (Model), mifepristone (Mife), quercetin high dose (QCT-H), and quercetin low dose (QCT-L). Except for the control group, rats received intraperitoneal injections of estradiol benzoate (0.5 mg/kg/day) and progesterone (4 mg/kg/week) for 6 weeks to induce UL formation. During the final 5 days of modeling, progesterone was administered daily in combination with estradiol benzoate.

From week 6 to 10, rats received daily oral administration of mifepristone (2.25 mg/kg/day), quercetin high dose (100 mg/kg/day), or quercetin low dose (50 mg/kg/day). Control and model groups received equal volumes of saline. To maintain the leiomyoma model, estradiol benzoate (0.5 mg/kg) was administered intraperitoneally every other day and progesterone (4 mg/kg) once weekly during the treatment period. Body weight was recorded weekly. At the end of the experiment, rats were fasted overnight and anesthetized prior to tissue collection (Figure 5A).

### 4.12. Histological Staining (H&E and Masson’s Trichrome)

Rat uterine tissues were fixed in 4% paraformaldehyde, dehydrated through graded ethanol, cleared in xylene, embedded in paraffin, and sectioned into 4 μm thick sections. For hematoxylin and eosin (H&E) staining, sections were deparaffinized, rehydrated, and stained with hematoxylin followed by eosin to evaluate histopathological changes. For Masson’s trichrome staining, sections were stained according to the manufacturer’s instructions to assess collagen deposition and fibrosis. Stained sections were examined under a light microscope, and collagen deposition was quantified using ImageJ software (version 1.54g, NIH, Bethesda, MD, USA) as the ratio of collagen-positive area to total tissue area.

### 4.13. Immunohistochemistry (IHC)

Paraffin-embedded uterine sections (4 μm-thick) were deparaffinized and rehydrated. After antigen retrieval and blocking of endogenous peroxidase activity, sections were incubated overnight at 4 °C with primary antibodies against Ki67 (Cat. No. 28074-1-AP, Proteintech, Wuhan, China) and PCNA (Cat. No. ab29, Abcam, Cambridge, UK). Sections were then incubated with HRP-conjugated secondary antibodies and visualized using diaminobenzidine (DAB), followed by hematoxylin counterstaining. Images were captured using a light microscope, and the percentage of positively stained cells was quantified using ImageJ software (version 1.54g, NIH, Bethesda, MD, USA).

### 4.14. Statistical Analysis

Data are presented as mean ± standard deviation (SD). Statistical analyses were performed using GraphPad Prism 10.0 (GraphPad Software, San Diego, CA, USA). Differences among multiple groups were analyzed using one-way ANOVA followed by Tukey’s post hoc test, while two-way ANOVA was used for time-course data. A value of *p* < 0.05 was considered statistically significant.

## 5. Conclusions

In conclusion, the present study demonstrates that quercetin suppresses UL progression through modulation of METTL3-associated MAPK signaling. By targeting this regulatory axis, quercetin inhibits proliferative signaling, promotes apoptotic activation, and attenuates inflammatory responses in leiomyoma cells, with dose-dependent therapeutic efficacy observed in vivo. Importantly, our findings identify METTL3 as a potential upstream regulator linking epitranscriptomic control to MAPK-dependent cellular processes, providing new insight into the molecular mechanisms underlying UL pathogenesis. These results highlight METTL3-associated signaling as a potential therapeutic target and support the development of natural compound-based strategies for the treatment of uterine leiomyoma.

## Data Availability

The original contributions presented in this study are included in the article/supplementary Material. Further inquiries can be directed to the corresponding author.

## Supplementary Materials

The following supporting information can be downloaded at: https://www.mdpi.com/article/10.3390/ijms27104586/s1.

## Abbreviations

The following abbreviations are used in this manuscript:

UL	Uterine leiomyoma
QCT	Quercetin
Mife	Mifepristone
METTL3	Methyltransferase-like 3
MAPK	Mitogen-activated protein kinase
MEK	Mitogen-activated protein kinase kinase
p-MEK	Phosphorylated mitogen-activated protein kinase kinase
JNK	c-Jun N-terminal kinase
p-JNK	Phosphorylated c-Jun N-terminal kinase
ERK	Extracellular signal-regulated kinase
p-ERK	Phosphorylated extracellular signal-regulated kinase
p38	p38 mitogen-activated protein kinase
p-p38	Phosphorylated p38 mitogen-activated protein kinase
BAX	Bcl-2-associated X protein
BCL-2	B-cell lymphoma-2
c-caspase-3	Cleaved caspase-3
Ki67	Ki-67 antigen
PCNA	Proliferating cell nuclear antigen
IL-6	Interleukin-6
IL-8	Interleukin-8
IL-11	Interleukin-11
EdU	5-Ethynyl-2′-deoxyuridine
DAPI	4′,6-diamidino-2-phenylindole
H&E	Hematoxylin and eosin
PBS	Phosphate-buffered saline
FITC	Fluorescein isothiocyanate
PI	Propidium iodide
PCA	Principal component analysis
PVDF	Polyvinylidene difluoride
RT-qPCR	Real-time quantitative polymerase chain reaction
