# Supplementary material for: Quercetin Suppresses Uterine Leiomyoma Progression by Modulating METTL3-Mediated MAPK Signaling
Source: Int J Mol Sci. 2026 May 20;27(10):4586. doi: 10.3390/ijms27104586 (PMC13206870; doi:10.3390/ijms27104586)
Supplement: Supplementary file 1 [file ijms-27-04586-s001.zip › ijms-4272337-supplementary.pdf]

Figure S1. Flow cytometry gating strategy and control samples for Annexin V-FITC/PI apoptosis analysis.

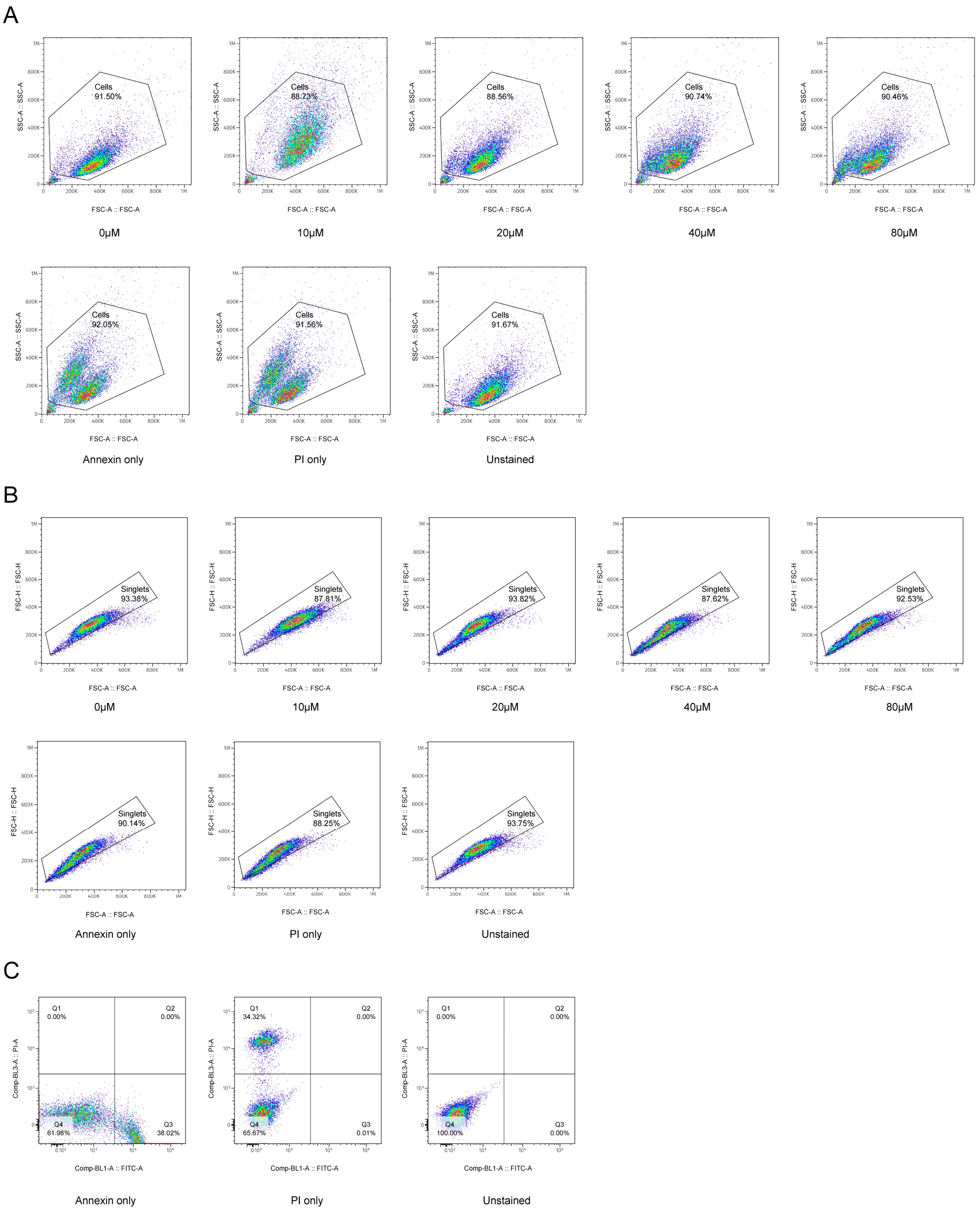

Figure S1. (A) Gating of the main cell population (“Cells”) based on FSC-A versus SSC-A. (B) Selection of single cells (“Singlets”) using FSC-A versus FSC-H. (C) Representative control plots including unstained, Annexin V-FITC single-stained, and PI single-stained samples used for compensation and quadrant setting. The same gating strategy was applied to all samples.

Table S1. Primers used for Real-Time Quantitative PCR.

Target Gene	Primer Sequences (forward)	Primer Sequences (reverse)
METTL3	TGGGGGTATGAACGGGTAGA	TGGTTGAAGCCTTGGGGATT
GAPDH	GGAAGCTTGTCATCAATGGAAATC	TGATGACCCTTTTGGCTCCC

Table S2. shRNA sequences targeting human METTL3

Oligo name	Target sequence (5′–3′)	Oligonucleotide sequence (5′–3′)
shMETTL3-1 (PSC126567)	GCTCAACATACCCGTACTACA	Forward: ccggGCTCAACATACCCGTACTACActcgagTGTAGTACGGGTATGTTGAGCtttttg<br>Reverse: aattcaaaaaGCTCAACATACCCGTACTACActcgagTGTAGTACGGGTATGTTGAGC
shMETTL3-2 (PSC126568)	GCTACCTGGACGTCAGTATCT	Forward: ccggGCTACCTGGACGTCAGTATCTctcgagAGATACTGACGTCCAGGTAGCtttttg<br>Reverse: aattcaaaaaGCTACCTGGACGTCAGTATCTctcgagAGATACTGACGTCCAGGTAGC
shMETTL3-3 (PSC126569)	GCAGTTCCTGAATTAGCTACA	Forward: ccggGCAGTTCCTGAATTAGCTACActcgagTGTAGCTAATTCAGGAACTGCtttttg<br>Reverse: aattcaaaaaGCAGTTCCTGAATTAGCTACActcgagTGTAGCTAATTCAGGAACTGC
